# Intraoperative Sodium-Fluorescence Imaging in Peripheral Nerve Sheath Tumors (PNST)—A New Additional Promising Diagnostic Tool

**DOI:** 10.3389/fonc.2021.655392

**Published:** 2021-03-09

**Authors:** Maria Teresa Pedro, Nadja Grübel, Gregor Durner, Andrej Pala, Christian Rainer Wirtz, Ralph Werner Koenig

**Affiliations:** Klinik für Neurochirurgie, Medizinische Fakultät, Universität Ulm, Ulm, Germany

**Keywords:** schwannoma, neurofibroma, malignant peripheral nerve sheat tumor, lymphoma, biopsy, sodium-fluorescein

## Abstract

**Background:** Through the development and implementation of specific fluorophore filters to microscopes in 2012, sodium fluorescein (SF) is currently experiencing a remarkable renaissance in neurosurgery. The present study examines its intraoperative application during surgical removal of peripheral nerve sheath tumors (PNST) and metastases.

**Methods:** This single-center study includes 10 cases of benign and malignant tumors as well as metastases of peripheral nerves (in total 11 PNST). Their surgical resections were all performed under microscope-based fluorescence with SF, which was administered intravenously (0.5–1.0 mg/kg body weight) during anesthesia induction. Microsurgical tumor removals were filmed and the collected data were retrospectively analyzed via ImageJ.

**Results:** Microsurgical tumor preparation was possible under the usage of fluorophore filter. In seven histological confirmed schwannoma (*n* = 6 patients) tissue differentiation between tumor mass and not involved fascicles was statistically significant for the colors green and red. Schwannoma maximum mean for green reached 254.7 pixel and 179.4 pixel for red, whereas passing healthy fascicles revealed a maximum mean for green 94.91 and for red 120.76 pixel. One case of neurofibroma achieved lower amount of pixel. Similar to schwannoma, the two MPNST cases showed a strong homogeneous fluorescence (max. mean green 215 pixel and red 124.51) involving the whole nerve segment. Subcutaneous tumor remnants were visualized and therefore resected. Via fascicular nerve biopsy a B-cell lymphoma of the tibial nerve could be detected. SF led to variable stain intensities in single fascicles. The resected fascicle revealed a max mean green of 100.54 pixel, whereas surrounding fascicles came up with max. mean green of 63.0 pixel.

**Conclusions:** Intraoperative SF visualization for PNST is feasible and of low risk. During resection of benign PNST, enhanced tissue differentiation between affected and not affected nerve segments is very useful. Tumor remnants can be detected safely and effectively. Its application during resection of malignant PNST is limited. Due to the infiltrative nature of those tumors, intraneural tissue differentiation is not possible. “Fluorescence-guided” biopsy can be regarded as an additional advantage in PNST surgery. Due to the encouraging experience in our institution SF was established as standard visualization tool in PNST surgery.

## Introduction

### Sodium-Fluorescein

Although an American neurosurgeon was the first one to describe the application of Sodium fluorescein (SF) in brain surgery in 1948 (1), this fluorphore medium fell into oblivion.

Only in 2003 and 2010 Shinoda and Okuda published their results in brain tumor surgeries under the usage of SF (2, 3). Their patients received intravenously a dosage of 20 mg/kg bodyweight which led to a distinct tumor mass fluorescence under white light.

However, since 2012 through the development and implementation of specific fluorophore filters to microscopes (e.g., YELLOW 560 nm filter; Zeiss Meditec, Oberkochen, Germany) its dosage has been significantly reduced to 2–5 mg/kg bodyweight. By this means, until today SF has experienced a tremendous renaissance in neurosurgery (4–8).

### Peripheral Nerve Sheath Tumors

Since 2013 the WHO classification has no longer assigned peripheral nerve sheath tumors to tumors of the central nervous system, but to tumors of the soft tissues and bones (9). Schwannoma and neurofibroma each make up about 5% of the soft tissue tumors (10), both rare entities are considered as benign. Their surgical enucleation should not lead to neurological deterioration. But depending on location, size and tissue consistency its surgical preparation may be demanding.

Malignant peripheral nerve sheath tumors (MPNST) are aggressive infiltrative growing tumors leading to a remarkable quickly emerging neurological impairment and resting pain. Until now the only effective therapy is surgical resection with wide negative margins (11). While post-operative radiation is recommended, the efficacy of chemotherapy remains unclear (11, 12).

The present study examines our experience and benefits of intraoperative SF application during surgical removal of different peripheral nerve sheath tumors (PNST) and metastases.

## Methods

### Study Group, Dosage, and Technical Equipment

This present single-center study includes 10 cases of benign and malignant tumors as well as metastases of peripheral nerves (in total 11 PNST). Patients over ≥ 18 years of age and with suspicion of PNST in MRI were included, whereas patients with history of renal insufficiency or SF intolerance were excluded. Their surgical PNST resections, respectively, biopsies were all performed by one surgeon under microscope-based fluorescence with SF (YELLWO 560 nm filter, Zeiss Meditec, Oberkochen, Germany) between August 2017 and September 2020 (Table 1). SF (Fluorescein Alcon 10%, Freiburg, Germany) was administered intravenously (0.5–1.0 mg/kg body weight) directly during anesthesia induction.

**Table 1:** Study group overview.

**Patient**	**Age (years); Sex**	**Affected nerve**	**Histology**	**Resection (complete/biopsy)**	**Surgeon's opinion**
1	24 yrs; female	Median nerve	Schwannoma	Complete	Helpful
2	45 yrs; female	Sural nerve	Schwannoma	Complete	Helpful
3	50 yrs; male	Tibial nerve	Schwannoma	Complete	Helpful
4	37 yrs; female	Median nerve	Schwannoma	Complete	Helpful
5	48 yrs; male	Peroneal nerve	2x schwannoma	Complete	Helpful
6	41 yrs; female	Peroneal nerve	Schwannoma	Complete	Helpful
7	45 yrs; male	Median nerve	Neurofibroma	Complete	Helpful
8 Illustrative case	77 yrs; female	Cutanaeus nerve paraspinal	MPNST	Complete	Very helpful
9 Illustrative case	55 yrs; male	Ulnar nerve	MPNST	Biopsy	Not helpful
10	71 yrs; male	Tibial nerve	B-cell lymphoma	Biopsy	Helpful

Since the application of SF is still restricted to ophthalmologic indications in Germany, the neurosurgical usage was off- label. Therefore, informed written consents were obtained from all patients emphasizing its intraoperative use. As individualized treatment, this study is in accordance with the ethical principles that are reflected in the Declaration of Helsinki.

### Image Evaluation

Microsurgical preparation was filmed and the collected data were retrospectively analyzed via ImageJ, an open source Java image processing platform (Version 1.51, National Institutes of Health, Bethesda, Maryland, USA) (13). Therefore, regions of interest (ROI) were set on the tumor mass and on inconspicuous nerve segments. For each ROI the quantity of the complementary colors red and green were evaluated.

### Data Analysis

For statistical analysis, SAS software version 9.4 (SAS Institute Inc., Cary, North Carolina, USA) was applied. First of all, frequencies, mean values as well as position and dispersion measures were calculated. For the latter, the representation was done with box plot diagrams. The test for normal distribution was carried out with the Shapiro-Wilk test. The non-parametric Mann-Whitney-U test was used to compare interval-scaled variables and the central tendency. The tests were carried out on both sides. *P* < 0.05 (α = 0.05) were rated as statistically significant with an error probability of five percent.

## Results

### General Results

The present cohort consists of each 5 male and female patients ranging in age from 24 to 77 years (mean age, 49 years). In total 11 PNST were histologically examined (7x schwannoma, 1x neurofibroma, 2x MPNST and 1x B-cell lymphoma). Complete tumor resection was performed in 8 patients, whereas 2 cases underwent fascicular biopsy (1x MPNST and 1x B-cell lymphoma). In 9 out of 10 cases (90%) the application of SF during tumor preparation was described as helpful by the surgeon (Table 1). No adverse events occurred under the applied dosage of 0.5–1.0 mg/kg bodyweight.

### Sodium-Fluorescein Results Correlating to Histopathology

**Figure 1:**
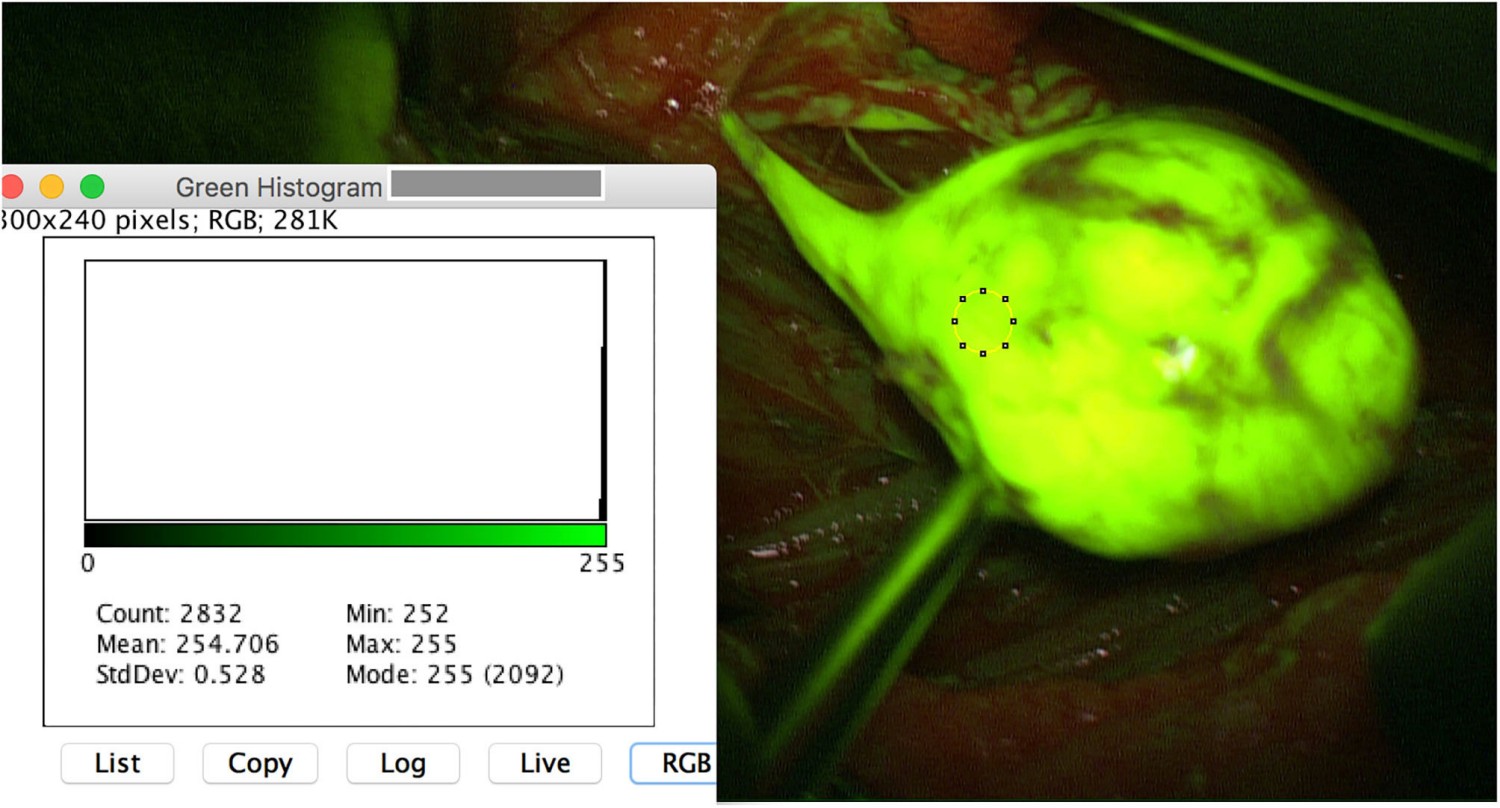
Intraoperative image of a schwannoma of the left tibial nerve after opening of the epineurium. The region of interest (ROI) is set on the tumor mass. ImageJ reveals a mean_*max*_ for the color green of 255 pixel with a standard deviation of 0.528. Note that the originating fascicle reveals a high yellowish stain.

Schwannoma reached a max. mean_green_ of 254.7 pixel and a max. mean_red_ of 179.4 pixel (Figure 1). In contrast, the passing healthy fascicles revealed a max. mean_green_ of 94.91 pixel and a max. mean_red_ of 120.76 pixel. The one neurofibroma case achieved a lower amount of fluorescence intensity. Its tumor mass max. mean_green_ reached 140.9 pixel and max. mean_red_ 76.2 pixel. Its not affected fascicles 88.5 (max. mean_green_) and 66.6 (max. mean_red_) pixel.

**Figure 2:**
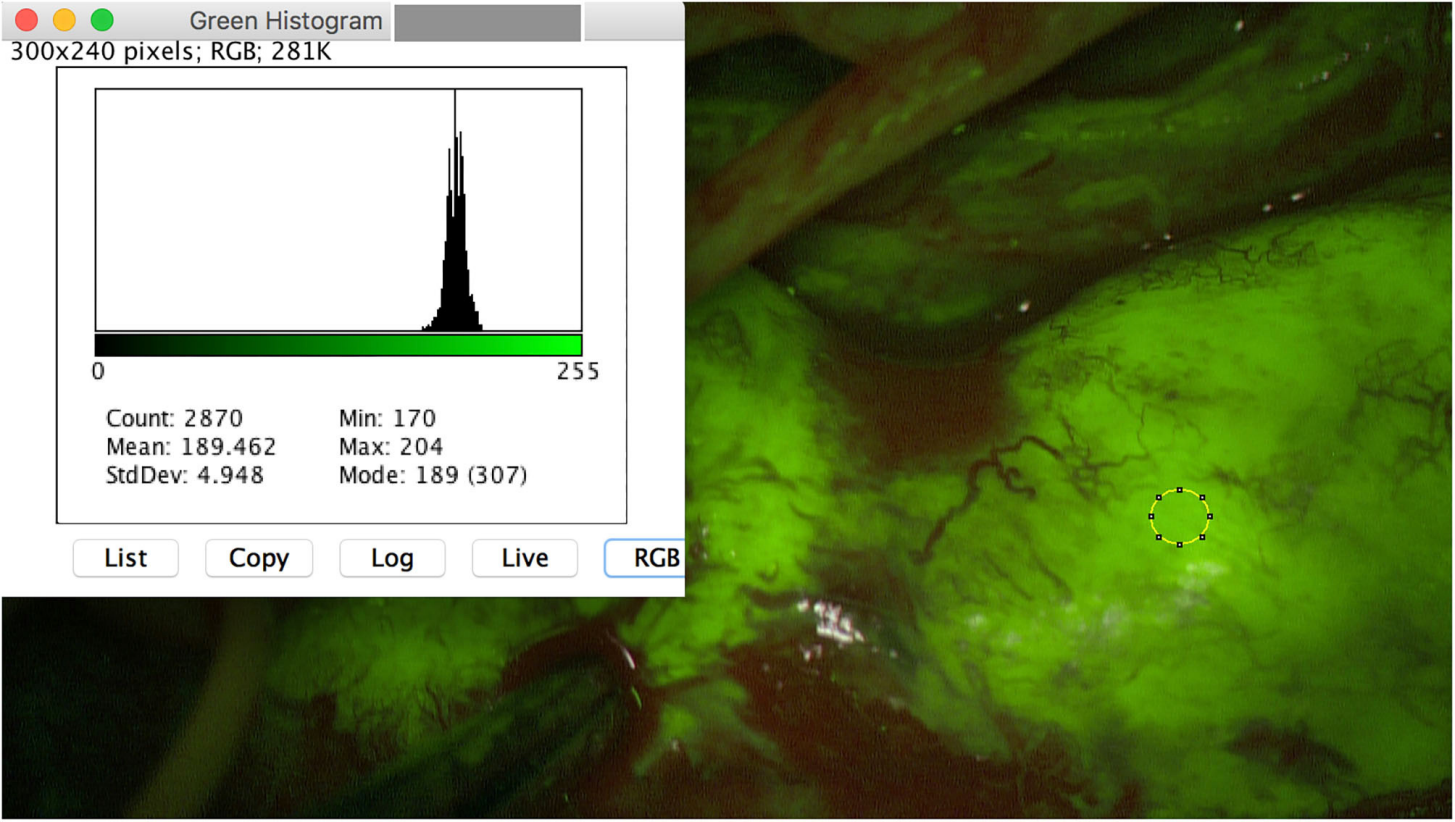
Intraoperative image of a MPNST of the right ulnar arm. A homogeneous fluorescence is seen. Mean_*max*_ for the color green is 204 pixel.

Similar to schwannoma, the MPNST achieved high SF intensities. One of both MPNST patients revealed an untypical MPNST anamnesis and tumor infestation, so that both cases are highlighted additionally as illustrative cases. MPNST max. mean_green_ was 215 and max. mean_red_ was 124.5 pixel. MPNST fluorescence was homogenous strong involving the whole nerve segment (Figure 2). Whereas, the surrounding subcutaneous tissue achieved lower values of 81.4 (max. mean_green_) and 73.4 (max. mean_red_). One patient suffering of a B-cell lymphoma in his medical history for 3 years, reported of a slowly progressing weakness of his right foot flexion. Since 2017 after chemotherapy he was already suffering a severe polyneuropathy. MRI showed a longitudinal positive contrast enhancement of a thickened tibial nerve. During tibial nerve biopsy, SF led to variable stain intensities in single fascicles. The resected fascicle revealed a max. mean_green_ of 100.54 and max. mean_red_ of 57.6 pixel. Surrounding fascicles came up with max. mean_green_ values of 63 and max. mean_red_ of 44 pixel.

**Figure 3:**
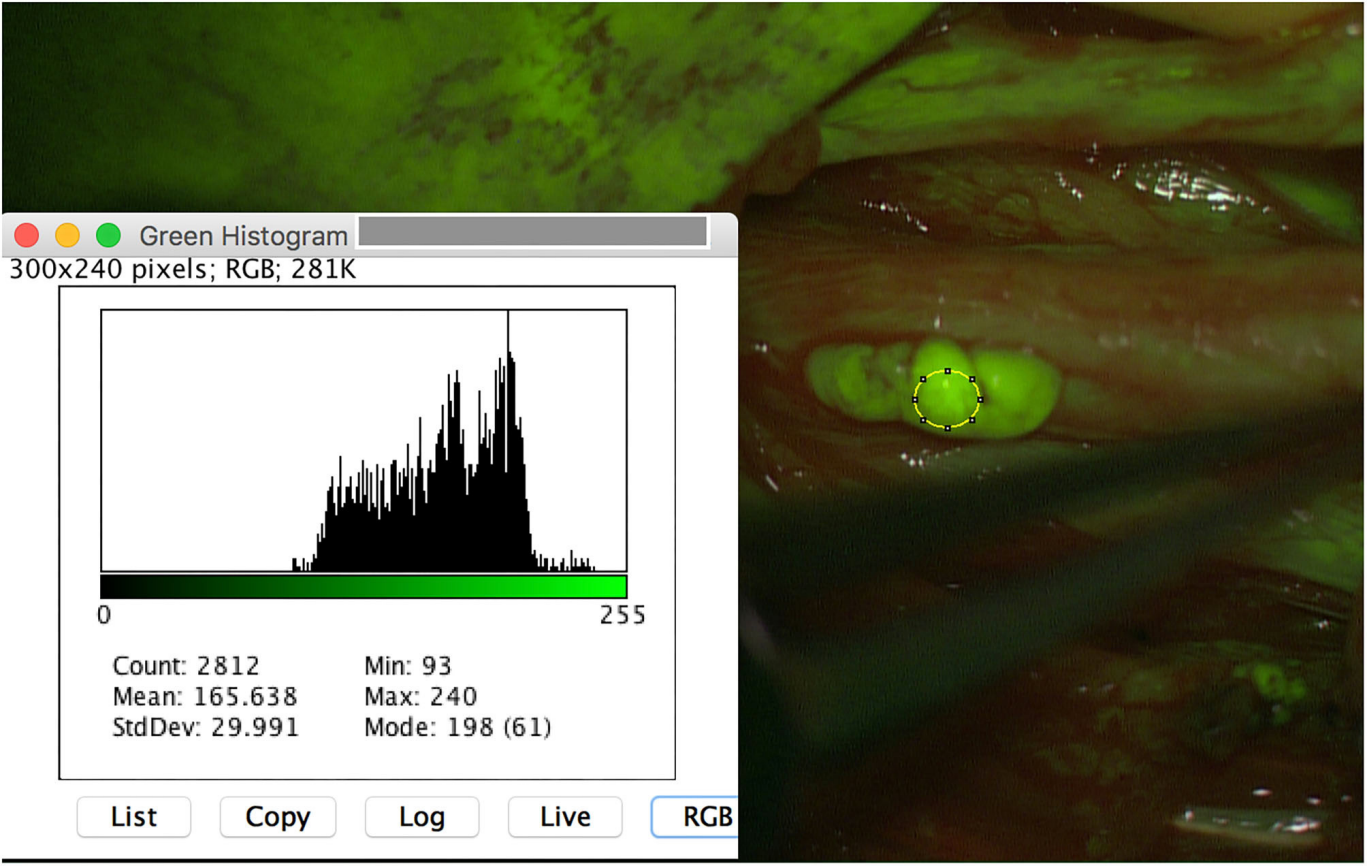
A small schwannoma (3 × 2 mm) of the left peroneal nerve is additionally detected with SF, achieving similar results of mean_*max*_ as the other schwannoma (240 pixel).

**Figure 4:**
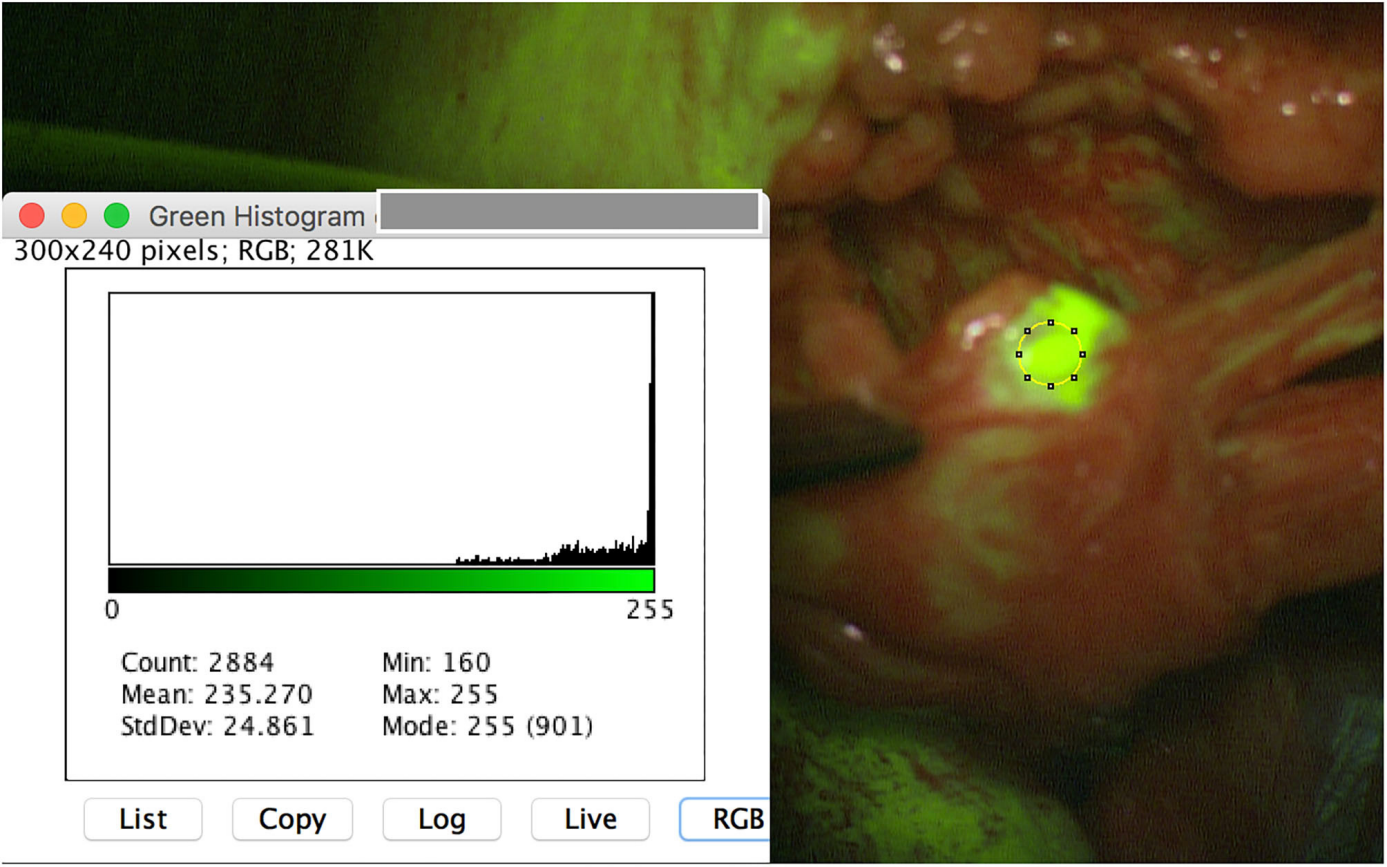
Example of visibility of fluorescent schwannoma remnant of right peroneal nerve.

Besides all these results, surgeon described the SF application as useful, since even very small PNST (for instance schwannoma 3 × 2 mm) were safely intraoperatively detected (Figure 3). Moreover, tumor remnants were securely depicted and resected (Figure 4).

**Figure 5:**
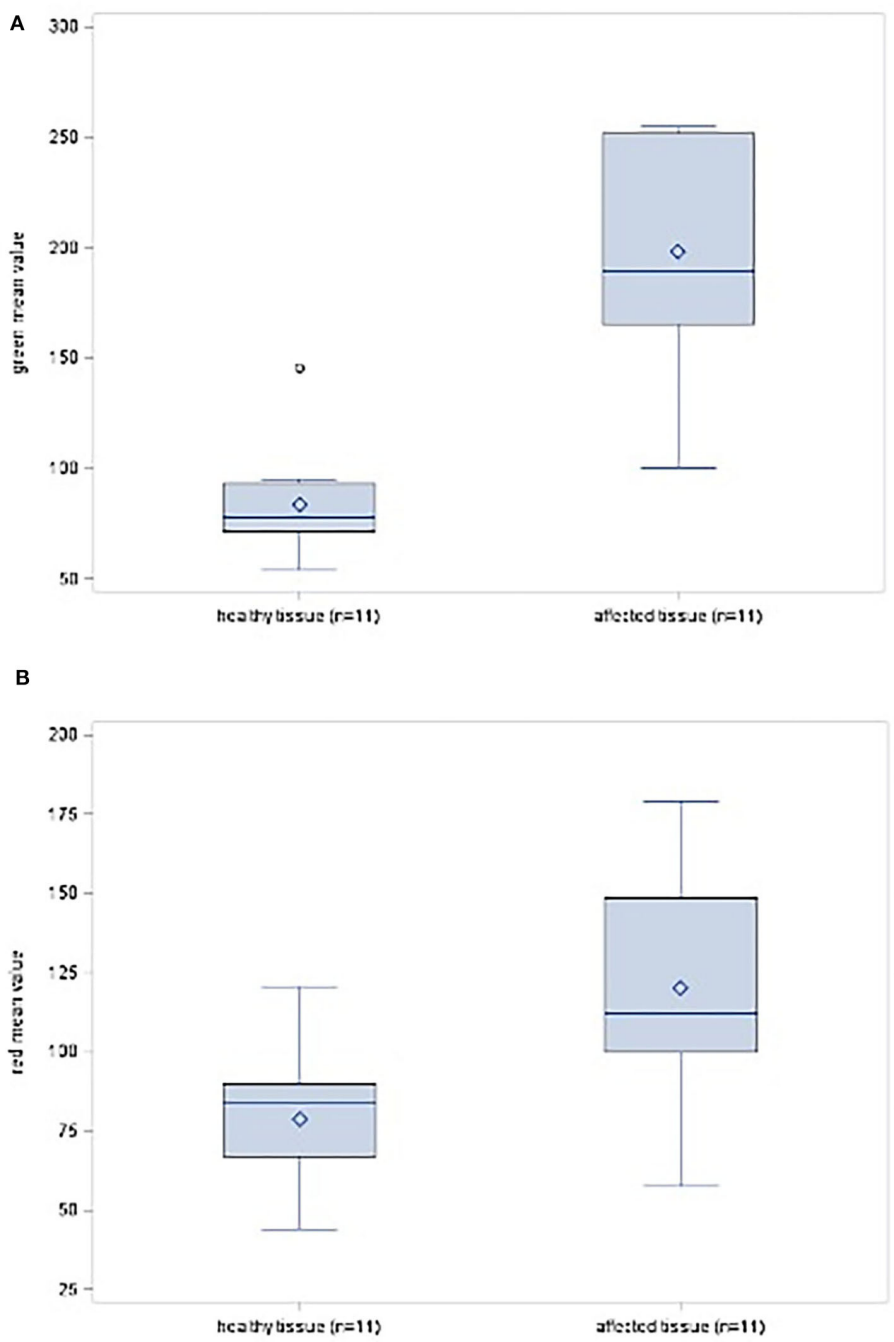
**(A)** Box-plot diagram showing the distribution scores for the color green in healthy and affected tissues. **(B)** Box-plot diagram showing the distribution scores for the color red in healthy and affected tissues.

In summary, in this present study group involving different PNST, tissue differentiation via SF was found to be statistically significant for the colors green (*p* = 0.0001) and red (*p* = 0.0071) (Table 2 and Figures 5A,B). Therefore, it is considered as a helpful intraoperative tool.

**Table 2:** Green and red mean differences between healthy tissue and affected tissue.

	**Mean** **±** **SD (Median) Min-Max**		
**Complementary colors**	**Healthy tissue (*n =* 11)**	**Affected issue (*n =* 11)**	***p-*Value**
Green mean value	83.36 ± 24.04 (78.00) 54.17–145.54	198.48 ± 49.97 (189.46) 100.54–254.71	0.0001
Red mean value	78.52 ± 20.13 (83.58) 44.02–120.76	120.16 ± 37.67 (111.94) 57.59–179.41	0.0071

*Counts are in pixel, p < 0.05 is determined as statistically significant*.

## Discussion

### Sodium-Fluorescein in Brain Surgery

The rediscovery and renaissance of SF in neurosurgery, started with its application during brain tumor surgery in the early 2000 (2). Indeed, currently many neurosurgical publications deal with SF. Through technical evolution of specific fluorescence filters and their implementation to microscopes in 2012, SF- visualization was significantly improved whereas its applied dosage was reduced. Subsequently in 2013 Schebesch et al. (14) published their first experience in sodium fluorescein-guided resections of malignant brain tumors. They found SF to be safe and feasible (14). At the same time, similar results were reported by an American group, they even extended SF's application to neurovascular malformations (7). Currently, SF is frequently used in surgeries of high-grade, low-grade gliomas, as well as meningioma or brain metastases (5, 6, 8, 15). Furthermore, SF is meanwhile applied in neuropediatrics (4, 16, 17). Due to this lower dosage of 2.0–5.0 mg/kg bodyweight no adverse events were reported in all those studies.

Nonetheless, the usage of SF in brain surgery has also been criticized by other study groups. In contrast to 5-aminolevulinic acid, SF reveals no glioblastoma cell-specific accumulation (18). After developing several *in vitro* and *in vivo* study models to investigate SF's biodistribution, Diaz et al. (18) came to the conclusion that SF accumulates in the extracellular space most likely due to the blood-brain-barrier dysfunction, similar to gadolinium contrast enhancement in MRI. Meanwhile, however, a recent study describes via SF a yellowish intraoperative fluorescence during resection of non-gadolinium positive brain tumors. Tumor detection and identification of its margins were possible in those gliomas. As common feature, they were all positive on F-fluoroethyltyrosine positron emission tomography (19).

In their most recent study, Bömers et al. (20) focused on surgeon reported usability of SF during glioblastoma surgery. Similar to the study results of Erdman et al. (16) and de Laurentis et al. (17) a high user satisfaction was observed (20). The identification of tumor location, margins and the secure microsurgical tumor resection under the SF filter was highlighted in all above-mentioned studies. Those results correlate well with our findings during PNST surgery.

### Sodium-Fluorescein in Peripheral Nerve Sheath Tumors (PNST)

Meanwhile, the application of SF is becoming a common tool for intraoperative tumor visualization, including intraneural ganglion cysts formations, in peripheral nerve surgery (21–24). According to our present results, it is a safe and useful method, which helps to define clearly tumor borders and hereby achieve gross total resection. Additionally, we were able to quantify these results using an imaging process platform (imageJ) and describe the different spectral ranges within different PNST and healthy tissue. Furthermore, in 10 out of 11 PNST resections, respectively, biopsies surgeon found SF to be helpful in this present analysis. As described in 2019, an application of low dose SF (0.5–1.0 mg/kg bodyweight) in an early stage (i.e., anesthesia induction), leads to a tissue differentiation between schwannoma and en passant fascicles. At the same time the standard principles of PNST microsurgical resection (i.e., direct motoric stimulation, EMG, NAPs, longitudinal dissection) have to be respected to preserve neurological function (21). Similar results were found by an Italian group in 2019. Vetrano et al. (22) examined 25 cases of PNST during surgery under SF. An optimal distinction between tumor and surrounding nerves was observed in 13 out of 14 schwannoma and in all 8 neurofibroma (22). Moreover, they emphasize the fact, that tumor remnants were not seen in 7 cases under white-light, but under SF (22). In contrast to those findings, a French group, examined 5 cases of schwannoma under the same circumstances. They did not see a benefit in between the tumor visualization under white-light and SF (25). Kalamarides stresses the need of intraoperative fascicular mapping and monitoring as stand-alone modality (25). All schwannoma were resected en-bloc, so that the need to depict possible tumor remnants did not arise. As seen in 2019 and in these present results, it is not always possible to detach schwannoma tissue from the healthy fascicles in toto, piecemeal technique has to be applied in some cases. Since a gross-total resection should be achieved, it is definitely reasonable to implement SF during schwannoma, respectively, neurofibroma resection (21, 22, 26).

MPNST are aggressive soft tissue sarcoma that account for ~5–10% of all cases (27). They mainly arise *de novo* in peripheral nerves or from neurofibroma (28).

Until now the only effective therapy and positive predictor of survival is surgical resection with wide negative margins (11, 12). While post-operative radiation is recommended, the efficacy of chemotherapy is controversially discussed (11, 12). As illustrated below, two MPNST patients were operated under the usage of SF. In one case, the whole ulnar nerve achieved a strong homogeneous yellowish stain, so that an intraneural tissue differentiation was not seen under the SF filter. Its usage was considered as not helpful during biopsy. The other case, a MPNST manifestation most likely out of a paraspinal cervical nerve, was operated several times, since under white-light microscopy and frozen section analysis a gross total resection of the surrounding tissue was not achieved. During the third surgery, SF revealed fluorescence of subcutaneous tissue, so that by this means, a complete resection became possible. After radiotherapy the patient reveals no recurrence or metastases until now.

Despite MRI, ultrasound and electrophysiology, there are some cases of peripheral nerve lesions, that need fascicular biopsy for further diagnosis in order to determine the proper therapy (23, 29). Those lesions may look inconspicuous in white-light microscopy, so that the usage of SF has been additionally examined in those cases. As described in 2020, the intraoperative application of SF helps to visualize the most affected fascicles and to determine a target fascicle biopsy (23). In this study group one patient, suffering of a B-cell lymphoma, developed neurological deficits of the tibial nerve. MRI showed a longitudinal nerve enlargement and contrast enhancement. Oncologists set the indication for nerve biopsy. During surgery, different intensities of fluorescence were seen, after motoric mapping of the fascicles, surgeon decided to resect one strong fluorescent fascicle. Histopathology confirmed the manifestation of a B-cell lymphoma.

In summary, SF, applied in low dose and during anesthesia induction, is meaningful and easily feasible during PNST removals or biopsies. No adverse events occurred.

In future, direct implementation of an image processing platform tool into the microscope could additionally enhance the value of this technique. By this means, surgeons would immediately receive mean values of relevant regions of interest in real time. Thereby, differentiated intraoperative decision making would be further simplified and improved.

### Illustrative Cases

Patient no. 8 (Table 1), a 77 years old female, suffered of a walnut-sized tumorous subcutaneous nuchal formation. She had no pain or neurological deficits. By suspicion of an atheroma she underwent outpatient surgery. Histopathology revealed a MPNST, therefore, she was timely admitted to our clinical center. Up to this point of time, there were no pre-existing neurological diseases, no signs of neurofibromatosis or significant comorbidities.

**Figure 6:**
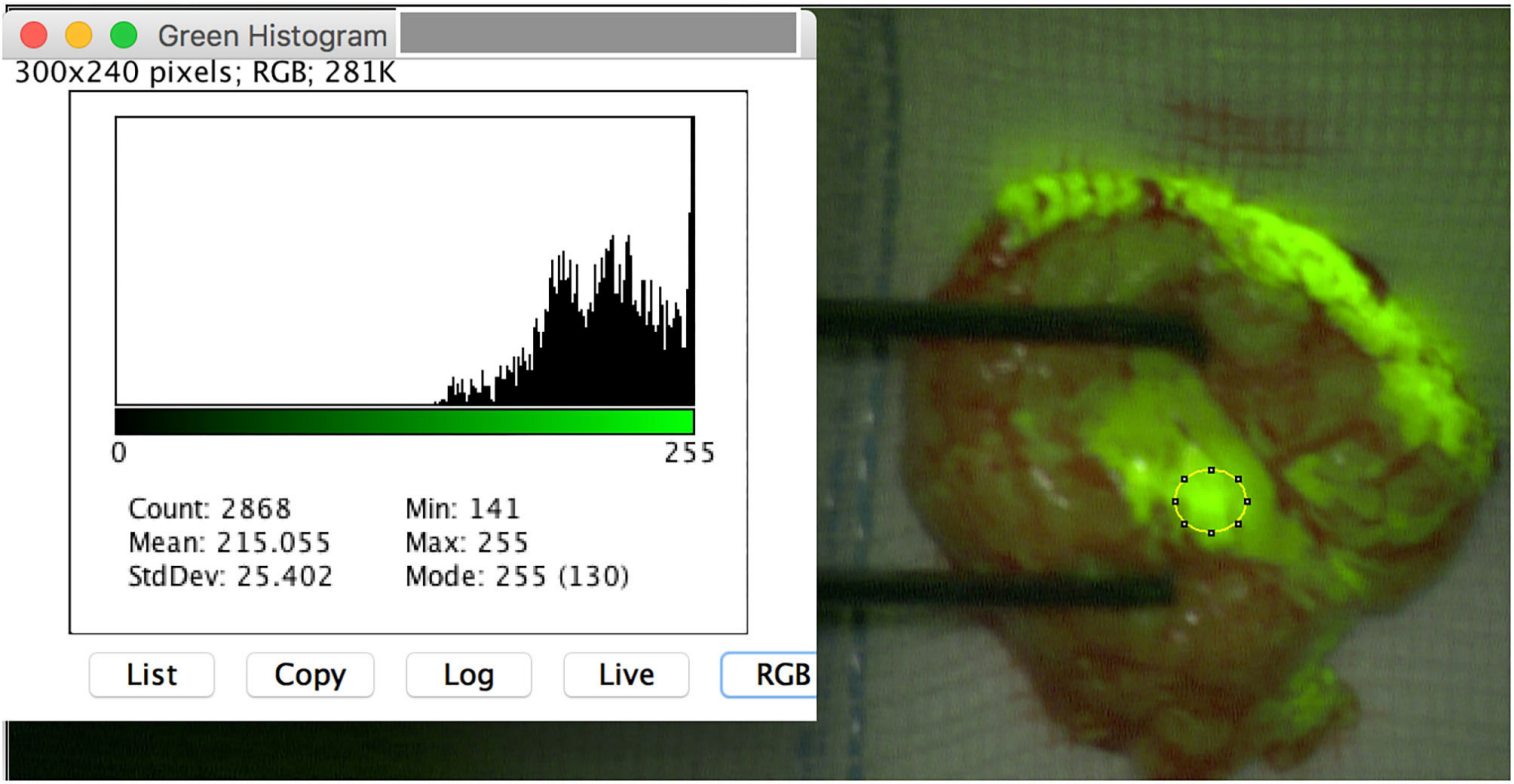
Subcutaneous resected tissue of MPNST of a paraspinal nerve (pat. n° 8). Yellowish tumor remnants became visible.

She was conducted to MRI and FDG-PET. Imaging depicted residual tumor mass and high accumulation of the surrounding tissue. Metastases were not detected. The second tumor resection was performed under white light. However, tumor free margins were not achieved according to the final histopathology results. As consequence, SF was applied during the third tumor resection. Subcutaneous tissue showed different degrees of fluorescence (Figure 6). Strong fluorescent areas were all resected. A complete tumor removal was finally confirmed by histopathology. A subsequent radiation followed. Until now, 18 months later, patient reveals no recurrence or metastases in the diagnostic follow-up.

**Figure 7:**
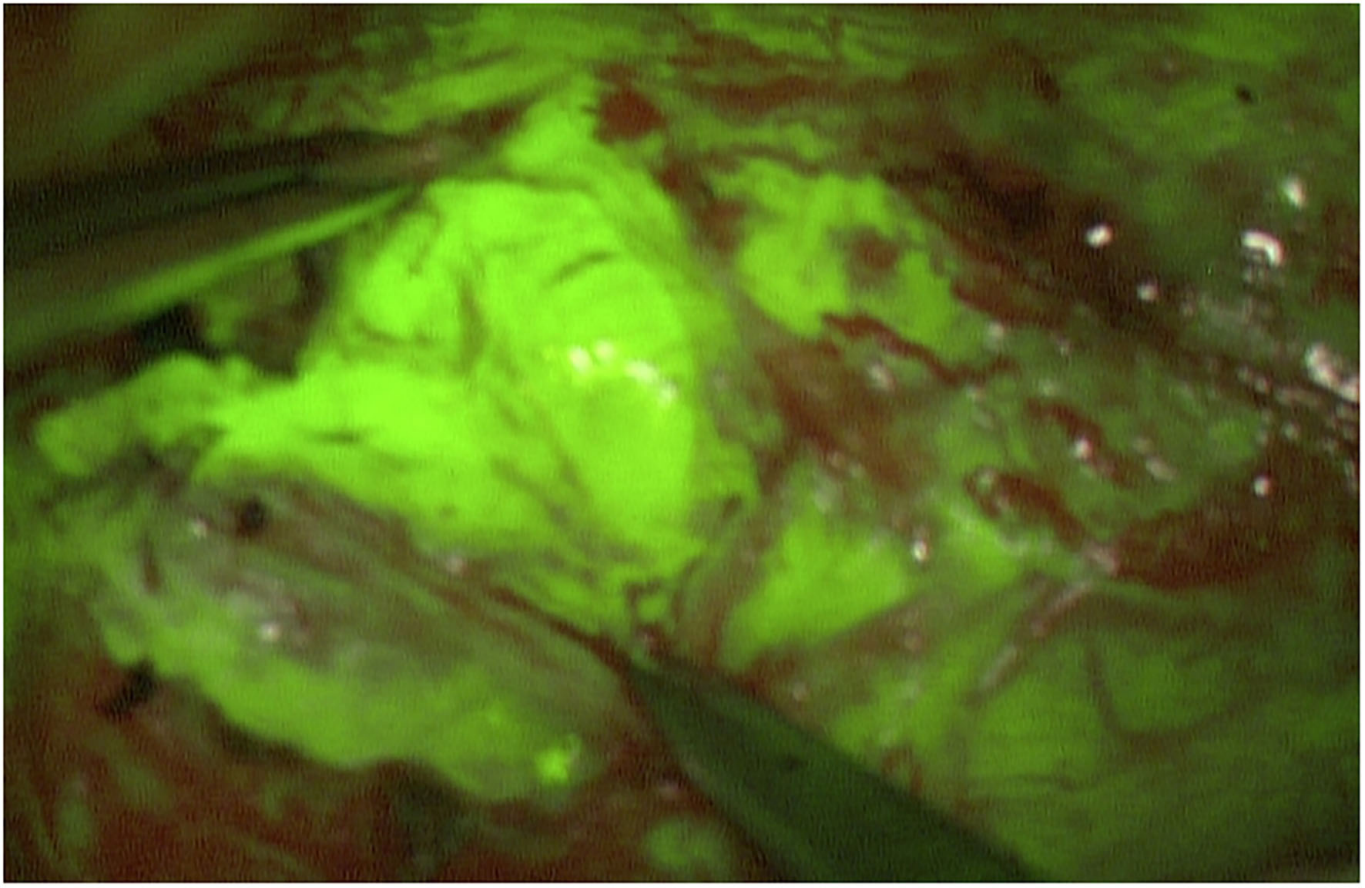
After opening of the epineurium of the ulnare nerve, the complete MPNST tissue reveals a strong homogeneous fluorescence. An intraneural tissue differentiation is not possible via SF (pat. n°9).

Patient no. 9 (Table 1), a 55 years old male, suffered of slow progressing paralysis of his right hand for years. He described an hypesthesia of his ring and small finger, so that on suspicion of a Loge de Guyon entrapment syndrome, he underwent decompression of his right ulnar nerve. Three years later, pain and functional loss increased dramatically. MRI and ultrasound depicted a thickened, strong enhancing ulnar nerve along the complete upper arm. The patient revealed no signs of neurofibromatosis or schwannomatosis. There were no pre-existing diseases. Fascicular biopsy was performed under SF and direct motoric stimulation. After epineurotomy, all fascicles of the ulnar nerve showed a homogenous strong fluorescence. An intraneural tissue differentiation was not recognizable (Figure 7).

Histopathology resulted in MPNST. In accordance to the local tumor board, the right arm was amputated. Metastases were not detected at this point. Seven months later, the patient developed pulmonary metastases on both lungs. Besides surgical resection, he died 2 months later.

## Conclusion

During the resection of benign PNST, tissue differentiation between affected and not affected nerve segments became visible using SF. Tumor remnants were securely detected. This intraoperative visualization method seems to be a helpful tool for surgeons. In contrast, the usage in malignant PNST was limited. In those cases, an intraneural tissue differentiation was not possible, only the extraneural surrounding tissues revealed a far lower fluorescence than the MPNST. Concerning the sciatic infestation of lymphoma, SF was helpful in identifying affected nerve fascicles.

The intraoperative application of SF in PNST surgery has been established as standard visualization tool in the present clinical institution.

## Data Availability Statement

The raw data supporting the conclusions of this article will be made available by the authors, without undue reservation.

## Ethics Statement

Ethical review and approval was not required for the study on human participants in accordance with the local legislation and institutional requirements. The patients/participants provided their written informed consent to participate in this study.

## Author Contributions

MP principal investigator and data analysis. RK conception and ImageJ evaluation. CW study design and counselor. AP data analysis and study design. GD data analysis. NG ImageJ evaluation and study design. All authors contributed to the article and approved the submitted version.

## Conflict of Interest

The authors declare that the research was conducted in the absence of any commercial or financial relationships that could be construed as a potential conflict of interest.
